# A Prospective Phase II Study Evaluating Intraoperative Electrochemotherapy of Hepatocellular Carcinoma

**DOI:** 10.3390/cancers12123778

**Published:** 2020-12-15

**Authors:** Mihajlo Djokic, Maja Cemazar, Masa Bosnjak, Rok Dezman, David Badovinac, Damijan Miklavcic, Bor Kos, Miha Stabuc, Borut Stabuc, Rado Jansa, Peter Popovic, Lojze M. Smid, Gregor Sersa, Blaz Trotovsek

**Affiliations:** 1 Clinical Department of Abdominal Surgery, University Medical Centre Ljubljana, Zaloska 7, SI-1000 Ljubljana, Slovenia; mihajlo.djokic@kclj.si (M.D.); david.badovinac@kclj.si (D.B.); 2 Faculty of Medicine, University of Ljubljana, Vrazov trg 2, SI-1000 Ljubljana, Slovenia; rok.dezman@kclj.si (R.D.); miha.stabuc@kclj.si (M.S.); borut.stabuc@kclj.si (B.S.); rado.jansa@kclj.si (R.J.); peter.popovic@kclj.si (P.P.); lojze.smid@kclj.si (L.M.S.); 3 Department of Experimental Oncology, Institute of Oncology Ljubljana, Zaloska 2, SI-1000 Ljubljana, Slovenia; mcemazar@onko-i.si (M.C.); mbosnjak@onko-i.si (M.B.); 4 Faculty of Health Sciences, University of Primorska, Polje 42, SI-6310 Izola, Slovenia; 5 Clinical Institute of Radiology, University Medical Centre Ljubljana, Zaloska 7, SI-1000 Ljubljana, Slovenia; 6 Faculty of Electrical Engineering, University of Ljubljana, Trzaska 25, SI-1000 Ljubljana, Slovenia; damijan.miklavcic@fe.uni-lj.si (D.M.); bor.kos@fe.uni-lj.si (B.K.); 7 Clinical Department of Gastroenterology, University Medical Centre Ljubljana, Zaloska 7, SI-1000 Ljubljana, Slovenia; 8 Faculty of Health Sciences, University of Ljubljana, Zdravstvena pot 5, SI-1000 Ljubljana, Slovenia

**Keywords:** electrochemotherapy, hepatocellular carcinoma, open surgery, liver cancer, bleomycin

## Abstract

**Simple Summary:**

Electrochemotherapy is fast developing local ablative therapy that is nowadays being adapted also for the treatment of deep-seated tumors. The first reports demonstrated the feasibility and safety of the procedure in liver tumors, i.e., colorectal liver metastases and hepatocellular carcinoma (HCC). This prospective phase II study aimed to investigate the effectiveness and long-term safety of electrochemotherapy in the treatment of primary HCC not suitable for other treatment options. Electrochemotherapy was performed intraoperatively in 24 patients and proved to be effective, feasible, and safe with some procedure-related side effects. In the 32 treated tumors, a high response rate was achieved: 84.4% complete responses, 12.5% partial responses, and 3.1% stable disease with the durable response over 50 months in 78.0% of the treated nodules. Based on the current evidence, electrochemotherapy (ECT) can be considered as a treatment option for HCC in the cirrhotic liver not suitable for other curative treatment options according to the Barcelona Clinic Liver Cancer classification (BCLC) classification.

**Abstract:**

The aim of this clinical study was to investigate the effectiveness and long-term safety of electrochemotherapy as an emerging treatment for HCC in patients not suitable for other treatment options. A prospective phase II clinical study was conducted in patients with primary HCC who were not suitable for other treatment options according to the Barcelona Clinic Liver Cancer classification. A total of 24 patients with 32 tumors were treated by electrochemotherapy. The procedure was effective, feasible, and safe with some procedure-related side effects. The responses of the 32 treated nodules were: 84.4% complete response (CR), 12.5% partial response (PR), and 3.1% stable disease (SD). The treatment was equally effective for nodules located centrally and peripherally. Electrochemotherapy provided a durable response with local tumor control over 50 months of observation in 78.0% of nodules. The patient responses were: 79.2% CR and 16.6% PR. The median progression-free survival was 12 months (range 2.7–50), and the overall survival over 5 years of observation was 72.0%. This prospective phase II clinical study showed that electrochemotherapy was an effective, feasible, and safe option for treating HCC in patients not suitable for other treatment options.

## 1. Introduction

The incidence of primary liver cancer is increasing worldwide, with a third of every cancer-related death being caused by liver tumors. Hepatocellular carcinoma (HCC) is the most common type of liver tumor, accounting for more than 90.0% of all liver tumors, followed by cholangiocarcinoma (CHC) accounting for 8.5% [1,2,3,4].

Two established options for the curative treatment of HCC are radical liver resection and liver transplantation [5,6]. The 5-year survival rates of these two methods can reach up to 70.0%, and both options can also provide good long-term survival [7,8]. Radiofrequency ablation (RFA) and microwave ablation (MWA) may also be used with curative intent for tumors smaller than 3 cm [9]. Unfortunately, fewer than 20.0% of HCC patients are eligible for these treatments [7,8]. If radical treatment is not indicated, transarterial chemoembolization (TACE), transarterial radioembolization (TARE), and other methods prolong the survival of these patients and can be used as a bridging therapy for later liver transplantation [10]. However, the survival of these patients remains poor [11]. Therefore, new treatment modalities for these patients with palliative or even curative intent are needed. In the perspective of the potential immunotherapy approach in the treatment of HCC, the greatest potential is in combination with ablative techniques, either thermal like RFA or MWA, or non-thermal such as electrochemotherapy (ECT) [12].

Electroporation-based treatments are becoming an important local treatment option for liver tumors [13,14,15], while ablation of liver tumors with irreversible electroporation (IRE) has already been well established, ECT, which is also a treatment for HCC, is lagging behind [16]. ECT is an ablative technique that utilizes electroporation for enhanced drug delivery into cells, where the drug exerts an enhanced cytotoxic effect in the electroporated area [16].

Based on its effectiveness for cutaneous tumors, ECT is now being developed and has been shown to be feasible, safe, and effective for deep-seated tumors, such as sarcomas, and liver tumors [17]. Our pilot study using ECT for the treatment of HCC proved its feasibility and safety [13], whereas a recent prospective phase II study on colorectal liver metastases clearly showed its long-term effectiveness as well [14]. These studies were performed intraoperatively, although the feasibility of the percutaneous approach has also been demonstrated [18]. Therefore, the most suitable drugs used for this treatment approach are hydrophilic drugs with low diffusion or lack of transport systems across the plasma membrane. In this category, bleomycin is the most suitable drug, which was demonstrated to have a several thousand-fold increase in cytotoxicity due to electroporation. Therefore, in vivo low drug concentrations are needed to obtain good antitumor effectiveness without systemic side effects [19]. Based on a previously reported pilot study, we continued with this prospective phase II study and evaluated the long-term safety and effectiveness of ECT for the treatment of HCC in patients not suitable for other curative treatment options according to the Barcelona Clinic Liver Cancer classification (BCLC) [9,13].

## 2. Results

### 2.1. Patients

According to the inclusion and exclusion criteria, 24 patients with 32 HCC lesions were treated in this prospective phase II clinical study (Table 1). ECT was performed during open surgery. The majority (70.8%) of patients were not suitable for other curative treatments according to the BCLC classification, while some were refractory to previous surgery or different local ablative techniques (Table 1). Based on the Child-Pugh classification, 9 patients had stage B liver cirrhosis and the remaining 15 had stage A. According to the American Society of Anesthesiologists (ASA) score, 6 patients had an ASA score of 2, and 18 patients had an ASA score of 3.

The treated lesions were predominantly solitary, although the maximum number in a single patient was 4 lesions. The average diameter was 2.5 cm, ranging from 0.8 to 4.5 cm as the longest diameter. The location of the tumors was either central (34.4%), defined when located in the vicinity of major blood vessels, or peripheral (65.6%), when located away from major vessels (Table 1).

### 2.2. Feasibility and Safety

ECT was feasible in all 24 patients. In group I, five patients with 8 lesions were previously treated with TACE and/or RFA, whereas, in group II, which consisted of 17 patients with 21 lesions, ECT was the primary treatment modality. Group III comprised 2 patients with 3 lesions who received ECT as bridging therapy to transplantation.

The treatment was performed either by electrodes with a fixed geometry (23 nodules) or with long single needle electrodes (9 nodules). The tumors located centrally were treated predominantly with electrodes with variable geometry (9/32 lesions), while those located peripherally were treated with electrodes with a fixed geometry (23/32 lesions). ECT was feasible, safe, and without immediate adverse events even in centrally located lesions in the proximity of major hepatic vessels (11/32 lesions).

**Figure 1:**
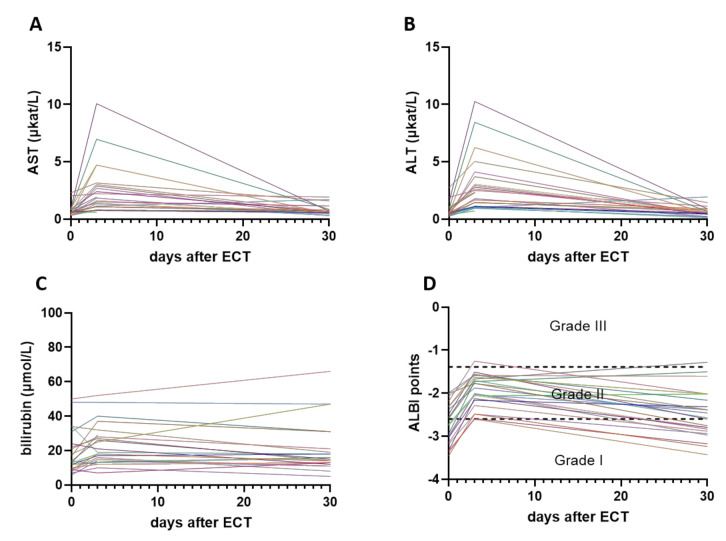
Laboratory values of Aspartate Aminotransferase (AST) (**A**), Alanine Aminotransferase (ALT) (**B**), and bilirubin (**C**) at the day of electrochemotherapy (ECT) (day 0), 3 days after the treatment, and at first follow-up, 30 days after the ECT. Each line represents one patient. Albumin-Bilirubin (ALBI) score for each patient at each time point was also calculated and ALBI grade was determined (**D**).

Intraoperative ECT had some procedure-related side effects. No serious adverse events according to the National Cancer Institute Common Terminology Criteria for Adverse Events (CTCAE) grade related to the procedure were observed within 24 h postoperatively in the intensive care unit level 1 (Table 2). The mean hospital stay was 5.5 days (ranging from 1–20 days). The perioperative period was defined as the period from the time of ECT to the time of first radiological evaluation, which was performed at a median of 30 days. During that time, we observed in 11 patients increased laboratory values of AST, ALT, and total bilirubin (Figure 1A–C), and those patients were classified according to Clavien-Dindo classification of surgical complications as grade I complication, but they did not need any other medical or interventional treatment (Table 2). Three days after the procedure, Albumin-Bilirubin (ALBI) score shifted to a higher grade in these 11 patients. After the first follow-up (median time 30 days), seven patients were downgraded to previous ALBI grade, the same as at day 0, prior to the procedure (Figure 1D). Two patients had wound healing problems (due to the development of ascites within the first two weeks) and were classified as Clavien-Dindo grade II. Two patients had Clavien-Dindo grade III complication, thus endoscopic retrograde cholangiopancreatography (ERCP) was performed in both of them because of the elevation of cholestasis enzymes 3rd and 4th day after the procedure, respectively. In both patients, only diagnostic ERCP was performed, without further or additional treatment.

ECG monitoring was performed, at least during the first 24 h after ECT. No signs of new-onset atrial and/or ventricular extrasystoles, myocardial ischemia, or an increased frequency of abnormal heartbeats in relation to the procedure were recorded. All 24 patients were followed on an outpatient basis.

### 2.3. Effectiveness

**Figure 2:**
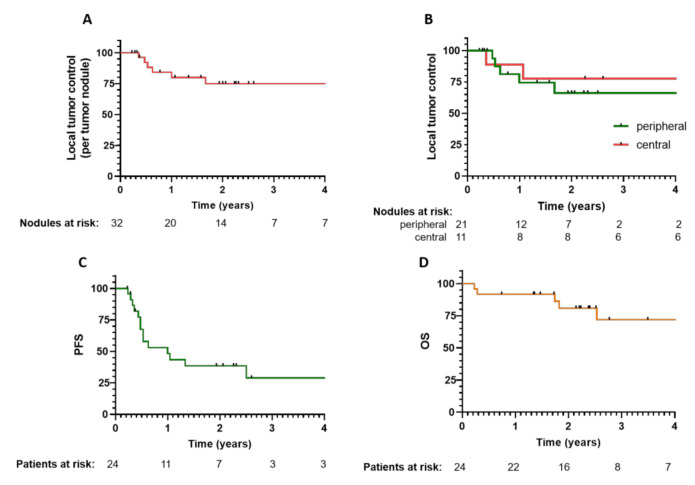
Local tumor control and response per patient to ECT treatment with the corresponding number at risk. Time to progression of the treated hepatocellular carcinoma (HCC) nodule (**A**). Local tumor control according to the tumor location, central or peripheral (**B**). Progression-free survival (**C**). Overall survival (**D**).

The response of the 32 treated tumors to ECT according to the modified RECIST (mRECIST) criteria was high: 27 tumors had a complete response (CR); 4 had a partial response (PR); 1 was stable disease (SD), and none progressed. The median observation time of the patients was 20 months (range 2.7–50.2 months). ECT was proven to induce a durable response rate since the local tumor control over 50 months of observation was 78.0% (Figure 2A).

**Figure 3:**
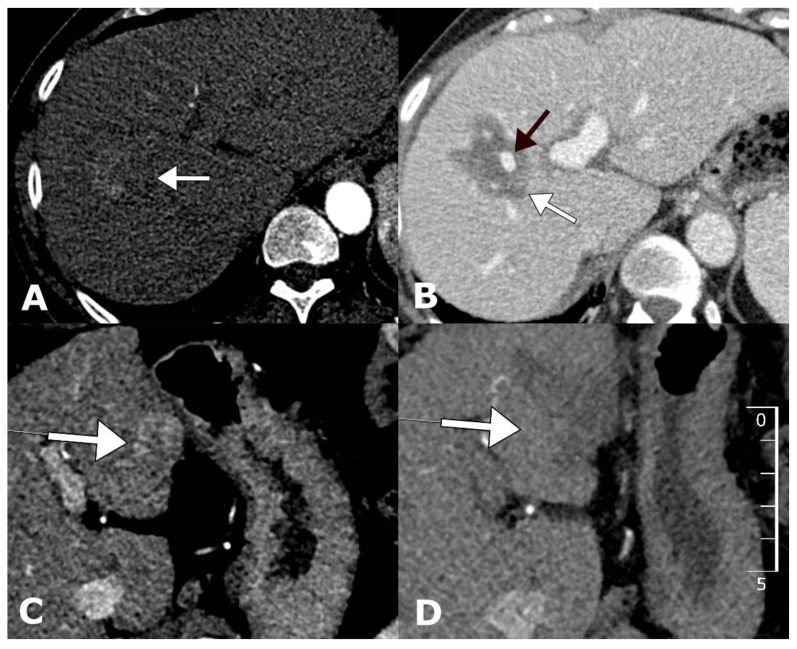
Examples of tumors located centrally and peripherally before and after treatment with ECT. Arterial-phase contrast-enhanced computed tomography (CECT) demonstrates centrally located HCC with a diameter of 31 mm located in the vicinity of a major portal vein branch (**A**). Follow-up CECT 1 month after ECT demonstrates a complete response with no tumor enhancement and a patent portal vein branch (**B**). Arterial-phase CECT demonstrates peripherally located HCC in liver segment III (**C**). Follow-up CECT 1 month after ECT demonstrates a complete response with no tumor enhancement (**D**).

The treatment was proven to be equally effective for tumors located centrally and peripherally since, at both locations, no significant difference in the level of tumor control was observed (*p* = 0.78). The centrally located tumors that were predominantly treated with electrodes for variable tumor geometry had a similar CR rate (81.8%) compared to those that were located peripherally and treated with electrodes with a fixed geometry (85.7%) (Figure 2B). Examples of CRs for tumors located centrally or peripherally are shown in Figure 3.

The median diameter of the treated tumors was 2.5 cm (range 0.8–4.5 cm). Usually, tumors with larger diameters have lower response rates than those with diameters smaller than 3 cm. Although the difference in the CR rate between tumors smaller than 3 cm in diameter (91.7%) and larger tumors (62.5%) was noticeable, the difference was not significant (*p* = 0.0854), which may be due to the low number of larger tumors treated with ECT.

The response rates after ECT per patient were 79.2% for CR and 16.6% for PR, which are lower than the response rates of nodules. This is due to the difference in the response of two or more tumors treated in the same patient.

In 15 patients (62.5%), the disease progressed in other parts of the liver or progressed extrahepatically. In this group of patients, 68.4% of the ECT-treated lesions remained in CR. The median progression-free survival of the ECT-treated patients was 12 months (range 2.7–50 months) (Figure 2C). The overall survival rate of patients was 72% at the 4-year observation time (Figure 2D).

## 3. Discussion

This prospective phase II clinical study showed ECT to be an effective, feasible, and safe option for treating HCC, with some procedure-related perioperative complications. CR to treatment was achieved in 79.2% of patients, and 84.4% of all treated lesions had a durable response.

Our study included patients who had previously undergone unsuccessful local ablative treatment and those with poor performance status, an unfavorable lesion position, or other contraindications to standard radical surgical treatment or bridging therapy to liver transplantation according to the BCLC classification. Among the 24 patients enrolled, perioperative complications were observed in 4, and even those were mild. These 4 patients experienced transient liver function failure with ascites, which later resolved spontaneously in two of the patients as well as in the other two after the administration of diuretic therapy. Regarding the poor performance status of some patients and other accompanying comorbidities, these complications bear little significance. Furthermore, ECT seems to be a feasible and safe treatment option even in such patients.

The effectiveness of ECT is comparable to the effectiveness of IRE. Studies on IRE have demonstrated an approximately 75.0% complete ablation rate of hepatic tumors that were surgically unresectable or had an unsuitable location for thermal ablation [20]. Our results are comparable to these previous results since we obtained an 84.0% CR rate per tumor and a 79.0% CR rate per patient. The results, therefore, are comparable to those of other ablative techniques, such as RFA. The study by Zimmerman et al. [21], based on a comprehensive literature search evaluating more than 200 treated patients, reported that the treatment effectiveness of IRE was similar to that of RFA, in tumors smaller than 3 cm in diameter. However, our results are limited in terms of the number of patients, therefore, further comparative studies are needed.

There are differences in the mode of action between IRE and ECT. IRE utilizes the application of electric pulses to damage the cellular architecture and physiology in order to induce cell death. Therefore, a higher number of pulses are needed to exert such action, which results in longer treatments [18]. Additionally, due to the higher number of pulses, IRE contains a nonnegligible thermal component, which can introduce additional safety concerns when treating targets near temperature-sensitive tissue, such as the central bile ducts [15,22,23,24]. In contrast, the advantage of ECT is the utilization of a smaller number of pulses for reversible electroporation of cells and the induction of apoptotic cell death with delayed action of cytotoxic agents and not electric pulses [18]. Therefore, ECT is a valid treatment option that may be utilized for HCC.

There seem to be different response rates of liver tumors to IRE and ECT. In IRE, the response of HCC is much better than the response of colorectal liver metastases [21]. Similarly, in our study, when compared to a previously reported study on colorectal liver metastases, the response rate of HCC was higher [14]. In our study, we obtained an 84.0% CR rate, whereas in the study on colorectal liver metastases, the CR rate was 63.0%. The comparison of these two studies is valid since both studies were performed with the same medical team, medical device, and treatment plan as well as with the same team for the evaluation of the response. The reason for the difference in the response is, therefore, not technical but rather due to tumor biology. Based on the knowledge of the mechanisms of action of ECT, we can speculate on the difference in drug pharmacology in tumors, which is due to the differences in the stroma and vascularization of tumors. According to tumor pathology, we know that HCC is better vascularized and that colorectal tumors are more fibrotic, and probably, the diffusion of drugs within colorectal liver metastases is hampered compared to HCC; therefore, there is less drug available for ECT [25,26]. Further studies are needed that take into account this biological aspect of tumors [27,28].

Tumor size seems to be an important prognostic factor of ECT effectiveness, as it is for other ablative techniques [14,29,30]. In lesions larger than 3 cm, the response to treatment is worse, and a CR is rarely achieved. However, in our study, we could not demonstrate the difference in the response rate. Although the percentage of CR in tumors larger than 3 cm was lower (62.5%) than in smaller tumors (91.7%), the difference was not statistically significant. The reason is the relatively small number of tumors (*n* = 8) larger than 3 cm in diameter compared to those smaller than 3 cm (*n* = 24). However, the difference in the response rates of tumors larger than 3 cm in diameter has been demonstrated in cutaneous tumors [29] and in the treatment of liver metastases, and it has been well established in other ablative techniques, especially RFA [9]. Therefore, specific attention has to be given to the treatment of tumors larger than 3 cm in diameter to observe treatment in the therapeutic window of 8–40 min after the infusion of bleomycin and to adequately cover the tumor with an electric field.

Electroporation-based treatments such as IRE and ECT provide safe and effective treatment for tumors located close to major liver vessels. These centrally located tumors, specifically in the hepatic hilum, can be approached with a treatment plan consisting of single, long needle electrodes placed with a variable geometry [13,14]. Numerical treatment planning for guidance is recommended to deliver an adequate electric field throughout the treated tumor and to assure safety margins in normal liver tissue. After the latest study in a pig liver model, we know that electroporation of larger vessels does not induce vessel damage or blood clotting [31,32]. The safety of such an approach was also demonstrated in phase II clinical study on the treatment of centrally located colorectal liver metastases by ECT [17,33]. Based on these data, we can recommend ECT for the safe and effective treatment of tumors located close to or adjacent to major hepatic vessels. Other ablating modalities such as MWA or multi-applicator RFA could also be effective for achieving the local tumor control in HCCs with perivascular locations [34]. These are, however, thermal ablation therapies and they pose the risk of injury to the vessels and major bile ducts [35]. Therefore, we believe that in the landscape of BCLC classification and when performed percutaneously, ECT could find its place among other ablative therapies for the treatment of HCC grade 0 to grade B and maybe also appropriate in the patients with slightly worse performance status (grade C, PS = 0–1).

The limitations of our study are the relatively low number of patients and intraoperative execution of the therapy. The study was designed with an intraoperative approach in order to verify the efficacy and safety of ECT. Now, based on the evidence of the efficacy, the translation to the percutaneous approach will be performed. The percutaneous approach will enable broader patients’ inclusion and provide a less aggressive approach, such as with IRE. We already successfully treated the first patient in a percutaneous approach [18].

ECT is a local treatment that does not prevent the outgrowth of new tumors outside the treatment zone. However, current investigations demonstrate that some local ablative techniques can induce a local immune response that can be boosted by immunotherapy. It has been demonstrated that ECT can induce local tumor vaccination that can be boosted by either immune checkpoint inhibitors or adjuvant immunotherapy [36,37]. Some studies have already demonstrated the potentiated local effect and some systemic effect of combined ECT and immune checkpoint inhibitors [12,38]. We also hypothesize that ECT combined with IL-12 gene therapy can transform ECT from local to locoregional or systemic therapy [39]. This has already been demonstrated in animal studies and in veterinary clinical studies but awaits testing and application in human oncology [40,41]. Such an approach, if proven effective, could prevent the outgrowth of other nodules and increase progression-free survival in ECT-treated patients.

## 4. Materials and Methods

### 4.1. Study Design

The study was designed as a prospective phase II study and was conducted at the University Medical Centre Ljubljana. The primary endpoint was to determine the efficacy of ECT based on radiological evaluation of the treated lesions, as measured by the modified RECIST (mRECIST) criteria [42,43]. The secondary endpoints were to evaluate the long-term safety in the treatment of HCC according to the Common Terminology Criteria for Adverse Events (CTC-AE) ver. 5.0 and Clavien-Dindo classification of surgical complications and response of ECT per patient. Prior to study initiation, approval from the National Medical Ethics Committee was obtained (21k/02/14). The study was registered at ClinicalTrials.gov (NCT 02291133). Patients were presented at multidisciplinary team meetings, which consisted of a surgeon, radiologist, and gastro-oncologist. Before inclusion into the trial, all patients signed written informed consent forms. ECT was performed according to the Standard Operating Procedures (SOP) for the treatment of cutaneous tumors and the associated modifications for the treatment of liver tumors [44,45].

### 4.2. Patients

In the current study, 24 patients with 32 lesions were enrolled from February 2014 to August 2019 based on the inclusion and exclusion criteria as previously described [13]. Briefly, the inclusion criteria were radiologically or histologically confirmed HCC in patients with a life expectancy of more than 3 months, patients older than 18 years with a Karnofsky performance status ≥70 or World Health Organization (WHO) <2, and patients who were not suitable for curative treatment according to the BCLC classification. The bilirubin-cut-off for study inclusion was 51.3 µmol/L. The patients did not meet any of the following exclusion criteria: (i) synchronous primary tumors, (ii) extrahepatic disease, (iii) poor performance status, (iv) clinically significant ascites, (v) impaired kidney function, (vi) allergic reaction to bleomycin, or (vii) exposure to cumulative bleomycin doses in excess of 400,000 IU.

Preoperative diagnosis of HCC was established by the typical radiological appearance (13 patients) according to the EASL–EORTC Clinical Practice Guidelines [46] or by histology (11 patients) [47]. Liver lesions were defined as peripheral or central. This definition was based on the relationship to major blood vessels. Peripheral lesions were not adjacent to major blood vessels, whereas central lesions were in close vicinity to the main hepatic or portal veins and the main hepatic arterial branches.

Regarding the indications for ECT and previous treatment modalities utilized, the patients were divided into three groups. The first group consisted of patients who had previously undergone local ablative treatment (TACE or RFA), which was unsuccessful or partially successful on follow-up according to mRECIST criteria (5 patients, Table 1). Each patient underwent at least two cycles of TACE before the decision about the treatment failure was made at the multidisciplinary team (MDT) meeting. The second group comprised patients with an unfavorable lesion position or other contraindications for radical surgery or liver transplantation based on the BCLC classification and with contraindication to the other ablative techniques due to the lesion location (17 patients). There were only two patients in the third group; they were offered ECT as a bridging therapy to liver transplantation that were not transplanted so far. One of the patients was BCLC stage 0, however, the tumor was embracing major hepatic vein and thus the patient was offered and treated with ECT.

Among included patients, 5 patients (21%) with a history of decompensation of cirrhosis were also included in the study; 3 patients (12.5%) with ascites, which reduced after diuretic therapy and 2 patients (8.5%) with bleeding from esophageal varices, which was treated by ligation before inclusion into the study. All patients, except one, were presented with cirrhosis; either post-hepatitis or alcoholic (ethylic). The patient who was not presented with cirrhosis was previously treated at a different institution due to tumor “origo ignota” in the right side of the liver, which was later histologically confirmed to be HCC. Right hepatectomy was performed at that time. Later, the patient was included in the current study. Due to the proximity of the new tumor nodule to the left hepatic vein, sole one, the MDT decided that ECT will be performed rather than MWA or RFA.

### 4.3. Treatment Procedure

Upper median laparotomy or median laparotomy extended with a right subcostal incision was used. Lesions were identified with intraoperative ultrasound, which also aided in the optimal positioning of the electrodes. The electrodes used for electric pulse delivery were either long needle electrodes (variable geometry) or hexagonal electrodes with a fixed geometry [37]. The choice of electrodes used was dependent on the location of the lesion. In liver lesions 3 cm or less beneath the capsule, ECT was performed with 3- or 4-cm-long needle electrodes with a hexagonal geometry. Longer needle electrodes (20 cm long, with an active part of 3 or 4 cm) with a variable geometry were used in deep-seated lesions. The hexagonal electrode had a maximum dimension in the plane of the electrodes of 14 mm; therefore, in order to achieve complete coverage of the target tissue, the electrodes were repositioned multiple times.

The long needle electrodes were positioned according to the pretreatment plan prepared individually for each patient and his or her specific tumor using previously developed procedures [13,48]. The plans were developed based on computed tomography and/or magnetic resonance scans obtained less than 30 days prior to treatment. The target lesions were segmented, and a feasible electrode insertion path was chosen by a physician. An appropriate voltage between each electrode pair was chosen by a gradient-based optimization algorithm, which ensured coverage of the tumor with electric fields above the reversible electroporation threshold (400 V/cm) while also keeping the predicted currents below 50 A, which is the hardware limit of the pulse generator [48,49].

**Figure 4:**
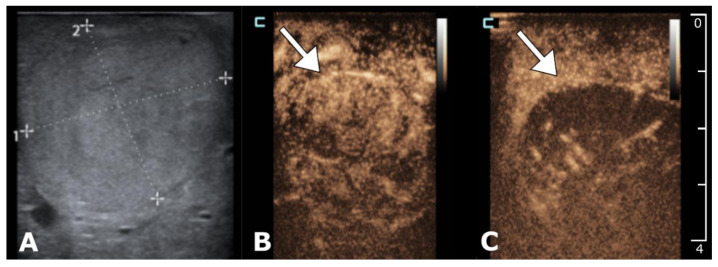
Intraoperative contrast-enhanced ultrasound (US) for tumor identification and treatment evaluation. B-mode ultrasound showing 24 mm subcapsular HCC in liver segment III (**A**). Contrast-enhanced ultrasound (CEUS) prior to ECT demonstrating a hypervascular tumor with early enhancement (**B**). CEUS two minutes post-ECT demonstrates reduced enhancement of the tumor with contrast due to the vascular lock effect (**C**).

An intravenous bolus of bleomycin (15,000 IU/m^2^, Bleomycin medac, Medac, Hamburg, Germany) was given to the patient after an intraoperative ultrasound confirmed the correct electrode placement. Eight minutes after bleomycin injection, electric pulses were delivered by Cliniporator^®^VITAE (IGEA SpA, Carpi, Italy). Eight electric pulses (electrodes with a variable geometry) or 24 (fixed geometry) electric pulses (each pulse was 100 µs long) were delivered to each pair of electrodes. Treatment was performed in an optimal window for ECT of 8–40 min after the intravenous injection of bleomycin as described in the updated SOP [44]. All pulses were synchronized with the absolute refractory period of the heart to prevent the electrical pulses from being delivered during the vulnerable ventricle period [49]. Intraoperative contrast-enhanced ultrasound (US) was utilized to evaluate the field covered with electroporation [50] (Figure 4). After treatment, patients were transferred to the ICU ward for monitoring.

### 4.4. Safety Assessment

Adverse events were assessed using the National Cancer Institute Common Terminology Criteria for Adverse Events (CTCAE) version 5.0. ECG monitoring was performed continuously during the surgical procedure.

### 4.5. Efficacy Assessment Based on Radiology

The lesions treated in the study were assessed before and after ECT by contrast-enhanced computed tomography (CECT) or with magnetic resonance imaging (MRI) using a distinct hepatocyte contrast (gadolinium ethoxybenzyl-diethylenetriaminepentaacetic acid—Gd-EOB-DTPA, Primovist, Bayer, Berlin, Germany). The treatment response of the treated lesion was evaluated by CECT or MRI using the mRECIST v1.1 criteria [42]. The evaluation was verified by a second blinded radiologist. Evaluations of both radiologists were in complete consensus. Patients were regularly followed for possible progression of the lesions and/or disease.

### 4.6. Statistical Analysis

Statistical analysis was performed with GraphPad Software (La Jolla, CA, USA). The log-rank (Mantel-Cox) test was performed on the Kaplan-Meier estimates. A chi-squared test was used for the statistical comparison of response according to the tumor location and size. A two-tailed *p*-value less than 0.05 was considered statistically significant.

## 5. Conclusions

In conclusion, this is the first prospective clinical trial to demonstrate the effectiveness, feasibility, and safety of ECT in the treatment of HCC. Based on the current evidence, ECT has its place in treating HCC in the cirrhotic liver not suitable for other curative treatment options according to the BCLC classification.

## Figures and Tables

**Table 1:** Patient and procedural characteristics.

Characteristics	Pts./Events	Percentage
Sex		
Male	17	71.0%
Female	7	29.0%
Age (years)		
Median	65.6	
Range	52–78	
Previous treatments of patients per study group		
GROUP I—Previous unsuccessful local ablative techniques	5	20.9%
RFA	1	4.2
TACE	2	8.3%
RFA + TACE	1	4.2%
MWA + TACE	1	4.2%
GROUP II—Only palliative treatment could be offered	17	70.8%
None	17	70.8%
GROUP III—Bridging therapy to transplantation	2	8.4%
None	1	4.2%
Surgery	1	4.2%
Child-Pugh score		
A (median score 5)	14	58.3%
score 5	9	
score 6	5	
B (median score 7)	9	37.5%
score 7	7	
score 8	2	
Esophageal varices	10	41.7%
BCLC stage		
0	1	4.2%
A	7	29.2%
B	16	66.6%
ASA score		
ASA 2	6	25.0%
ASA 3	18	75.0%
Number of tumors treated		
Total	32	
Average per patient	1.3	
Range	1–4	
Tumor size		
Average	2.5 cm	
Range	0.8–4.5 cm	
Type of electrodes used in ECT		
Fixed geometry	23	71.9%
Variable geometry	9	28.1%

RFA—radiofrequency ablation; MWA—microwave ablation; TACE—transarterial chemoembolization; BCLC—Barcelona Clinic Liver Cancer classification.

**Table 2:** Toxicity and treatment outcomes.

Characteristics	Pts./Events/Percentage	
Toxicity (CTCAE grade)		
ECT-related	0	
Non-ECT-related within 24 h	0	
Non-ECT-related after 24 h	4 (16.7%)	
Postoperative complications (up to 30 days after ECT) according to Clavien-Dindo Classification		
Grade I	11 (46%)	
Grade II	2 (8%)	
Grade III	2 (8%)	
Response to ECT/tumor (RECIST v1.1)		
Number of tumors	32	
CR	27 (84.4%)	
PR	4 (12.5%)	
SD	1 (3.1%)	
PD	0 (0%)	
Response to ECT/patient (RECIST v1.1)		
Number of patients	24	
CR	19 (79.2%)	
PR	4 (16.6%)	
SD	1 (4.2%)	
PD	0 (0%)	
Response according to tumor location	Central	Peripheral
Number of tumors	11 (34.4%)	21 (65.6%)
CR	9 (81.8%)	18 (85.7%)
PR	1 (9.1%)	3 (14.3%)
SD	1 (9.1%)	0 (0%)
Response according to tumor size	≤3 cm diameter	>3 cm diameter
Number of tumors	24 (75%)	8 (25.0%)
CR	22 (91.7%)	5 (62.5%)
PR	2 (8.3%)	2 (25.0%)
SD	0 (0%)	1 (12.5%)

CR—Complete Response, PR—Partial Response, SD—Stable Disease, PD—Progressive Disease.
